# Exploration of ABA Responsive miRNAs Reveals a New Hormone Signaling Crosstalk Pathway Regulating Root Growth of *Populus euphratica*

**DOI:** 10.3390/ijms19051481

**Published:** 2018-05-16

**Authors:** Conglong Lian, Kun Yao, Hui Duan, Qing Li, Chao Liu, Weilun Yin, Xinli Xia

**Affiliations:** 1 Beijing Advanced Innovation Center for Tree Breeding by Molecular Design, Beijing Forestry University, Beijing 100083, China; liancl00@163.com (C.L.); kunyao@bjfu.edu.cn (K.Y.); duanhui_star@163.com (H.D.); v_liqing@163.com (Q.L.); liuchao1306@163.com (C.L.); yinwl@bjfu.edu.cn (W.Y.); 2 National Engineering Laboratory for Tree Breeding, Beijing Forestry University, Beijing 100083, China; 3 College of Biological Sciences and Technology, Beijing Forestry University, Beijing 100083, China; 4 Key Laboratory of Genetics and Breeding in Forest Trees and Ornamental Plants, Beijing Forestry University, Beijing 100083, China

**Keywords:** *Populus euphratica*, abscisic acid, microRNA, high-throughput sequencing, hormone crosstalk, root growth

## Abstract

Abscisic acid (ABA) plays an important role in the regulation of plant adaptation, seed germination, and root development in plants. However, the mechanism of ABA regulation of root development is still poorly understood, especially through the miRNA-mediated pathway*.* Here, small RNA (sRNA)-seq and degradome-seq were used to analyze the miRNAs’ responsive to ABA in the stems and roots of *P. euphratica*, a model tree species for abiotic stress-resistance research. In total, 255 unique mature sequences, containing 154 known miRNAs and 101 novel miRNAs were identified, among which 33 miRNAs and 54 miRNAs were responsive to ABA in the roots and stems, respectively. Furthermore, the analysis of these miRNAs and their targets revealed a new hormone signaling crosstalk model of ABA regulation of root growth through miRNA-mediated pathways, such as peu-miR-n68 mediation of the crosstalk between ABA and the brassinosteroid (BR) signaling pathway and peu-miR477b mediation of the crosstalk between ABA and Gibberellic acid (GA) signaling. Taken together, our genome-wide analysis of the miRNAs provides a new insight into the mechanism of ABA regulation of root growth in *Populus*.

## 1. Introduction

Abscisic acid (ABA) plays a pivotal role in plant responses to biotic and abiotic stresses, such as pathogens, salinity, wounding, and water shortage [1]. As one of the most important phytohormones, the mechanisms of ABA signaling have been studied extensively for decades. Typically, ABA levels chang under different conditions and are modulated by the balance between ABA catabolism and biosynthesis [2], with 9-cis-epoxycarotenoid dioxygenase 3 (NCED3) being a vital enzyme for ABA biosynthesis [3] and ABA 8′-hydroxylases (encoded by a cytochrome P450 gene, *CYP707A*) being a key enzyme in ABA catabolism [4]. Furthermore, an overwhelming number of studies regarding ABA signaling components that regulate seed germination and stomata have been completed [5,6]. The most remarkable development of the ABA signaling pathway is the discovery of the ABA receptor, PYR/PYL/RCAR, which functions at the apex of a negative regulatory pathway that controls ABA signaling by inhibiting type 2C protein phosphatases (PP2Cs) [7,8]. Especially, ABA perception by RCARs/PYR1/PYLs plays an important role in the regulation of stomatal aperture, seed germination and vegetative and reproductive growth [9].

In addition, ABA is not only a stress signal in the regulation of stomatal aperture and gas exchange in plants leaves but is also required to fine-tune stem and root growth and development. ABA can be synthesized in the root, absorbed from soil water surrounding the root, and then delivered to the root from the shoots through the phloem [10]. The soil under drought and salt treatment could increase root hydraulic conductivity and increase the transport of the synthesized ABA from the root towards the xylem [11]. ABA plays a partial role in the maintenance of root growth [12] and controls shoot growth under water stress [13]. ABA also interacts with other phytohormones, such as gibberellic acid [14,15], brassinosteroids [16,17] and auxin [18] in regulating plant growth and development. However, the mechanisms of ABA signaling or interaction with other phytohormones relevant to the regulation of root growth is not clear.

MicroRNAs (miRNAs), a kind of single-stranded non-coding RNA, play a vital regulatory role in a variety of biological processes, such as development, stress, and hormone responses. Recent evidence indicates that some miRNAs are involved in ABA responses. The SnRK2 kinases, a core ABA signaling component play an important role in miRNA accumulation by phosphorylating miRNA the processing components, SERRATE (SE) and HYPONASTIC LEAVES (HYL1) in vitro [19]. RECEPTOR FOR ACTIVATED C KINASE 1(RACK1) scaffold proteins, which are involved in ABA responses, influence miRNA abundance via several distinct mechanisms involving direct interactions with SERRATE and are part of an AGO1 complex [20]. ABA-insensitive 5 (ABI5) is the target of a novel microRNA, Fan-miR73, in strawberries [21]. All of these studies have shown that ABA is involved in the formation of miRNA.

Some miRNAs have been shown to regulate root and stem growth and development. The miR160 family was first reported to be involved in root cap formation through the ARF10- and ARF16-mediated auxin signaling pathways in *Arabidopsis* [22]. Overexpression of miR164 reduces auxin signals for lateral root development [23] and also affects lignin metabolism by regulating transcription factors that control secondary growth and wood composition [24,25]. Furthermore, some miRNA families have been identified to be involved in the nutrition metabolism of roots, such as miR395 [26,27], miR398 [28], and miR399 [29,30]. Additionally, previous studies have shown that miRNAs are influenced by ABA in the roots and stems. The expression of miR169 is downregulated in response to ABA in maize roots [31]. miR842 and miR846 are regulated by ABA, as the cistronic miRNA pair is a product of alternative splicing in the roots of *Arabidopsis* [32]. In *Arabidopsis*, the overexpression of miR160 represses the expression of *ARF10* and *ARF16*, resulting in root tip defects with a tumor-like root apex and a loss of gravity sensing [22]. miR160 also reduces ABA sensitivity during seed germination [33]. However, the question of whether ABA is involved in root growth through the miRNA pathway remains to be further elucidated.

*Populus euphratica* adapts to stress conditions through plastic responses in the root [34]. It confers a high tolerance for drought, salinity, and cold, and it is a promising model system for studying the response to abiotic stress resistance in woody plants [35]. A number of reports have indicated that miRNA participates in abiotic stresses, such as drought [36], salt [37], and cold [38], in poplar trees. Based on our previous study on the miRNA response to ABA in the leaves of *P. euphratica* [39], a genome-wide analysis of miRNAs in response to ABA treatment was performed in the roots and stems. Furthermore, a new model of ABA regulation of root growth through the miRNA-mediated pathway was established. This genome-wide analysis can be expected to yield much valuable information and provide new insight into the molecular mechanism of ABA regulation of root growth.

## 2. Results

### 2.1. Overview of Small RNAs (sRNAs) Responsive to ABA in the Roots and Stems of P. euphratica

To systematically identify the responsive of miRNAs to ABA in the roots and stems of *P. euphratica*, eighteen sRNA libraries from stems (S) and root (R) of control (C), short-term (S), and long-term (L) ABA treatment, named CSs, SSs, LSs, CRs, SRs, and LRs, with three replicates were constructed from *P. euphratica* plants, with or without treatment with ABA solutions, and 10–17 million raw reads were acquired through high-throughput sequencing. After processing and filtering, the contaminant sequences with poor-quality reads and adaptor sequences, a total of more than 92% of the sequencing reads, remained. These clean sequencing reads were then mapped to the genome of *P. euphratica*, and the results indicated that in half of the libraries, more than 50% of the total sRNAs matched the genome of *P. euphratica* [40], as shown in Table 1.

**Figure 1:**
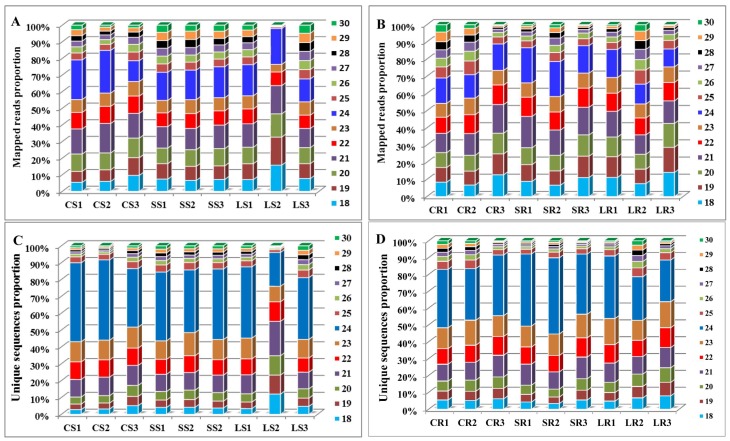
Length distributions of small RNAs identified in the roots and stem of *P. euphratica*. Size distribution of total mapped reads from the nine libraries of stems (**A**) and the nine libraries of roots (**B**). The distribution of stems (**C**) and roots (**D**) are based on the mapped unique reads.

The sRNAs with different sizes exhibited discrepant functions. Twenty-one nucleotide sRNAs usually function in posttranscriptional gene silencing, while 24 nt sRNAs mainly induce gene silencing mediated by heterochromatin maintenance or RNA-dependent DNA methylation [41,42,43]. There was no major difference in the size distribution of the high-throughput sequencing reads between the different groups of stems (Figure 1A) and roots (Figure 1B), and the largest number of sRNAs were 21–24 nt in length. Specifically, in the roots and stems, sRNAs with lengths of 24 nt were the most abundant and those of 21 nt were the second most abundant among all the libraries. This is consistent with the results of leaves in *P. euphratica* [39] and other plants, such as in *Lilium Regale* Wilson [44], and *Arabidopsis thaliana* [45]. The analysis of the size distribution of unique sequences of mapped reads revealed that the 24 nt length read was the most abundant, followed by the 23 nt class (Figure 1C,D). This result was consistent with previous studies in *Solanum tuberosum* L. [46]. Above all, these results suggest the existence of a complex and diverse sRNA population in *P. euphratica*.

### 2.2. Known miRNAs in P. euphratica Roots and Stems

**Figure 2:**
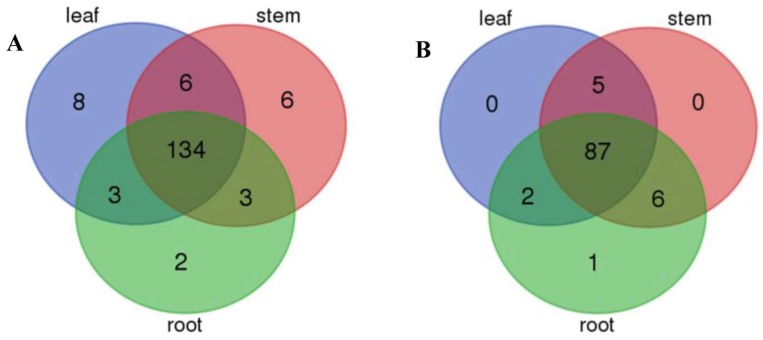
Venn diagram of the miRNAs identified in leaves, stems and roots of *P. euphratica*. Counts are based on unique sequences, and Venn diagrams of (**A**) known miRNAs and (**B**) novel miRNAs found in the different tissues.

Firstly, different types of sRNAs were identified in *P. euphratica* roots and stems, including miRNA, novel RNA, repeat-associated RNA, rRNA, snRNA, nat-sRNA, snoRNA, exons, intron, trans-acting sRNA, and other sequences, with the highest proportion of sRNAs located in the exons (Table S2). Here, 154 unique mature sequences, belonging to 71 known miRNA families, were identified in at least one of eighteen libraries (Tables S3 and S4). Among them, 98 unique miRNAs, belonging to 28 miRNA families, were conserved, and the other 56 miRNAs, belonging to 43 families, were poplar specific, with the exception of miRNA1446 found in *Gossypium raimondii* and miRNA475 found in *Medicago truncatula*. Compared to the leaf libraries of *P. euphratica* in the previous study [39], 11 more known miRNAs were found specifically in the roots and stems (Table 2). While 134 of the 162 known miRNAs are shared among the leaves, stems and roots, some known miRNAs showed tissue specificity (Figure 2A). There were 137 known miRNAs expressed in both the roots and stems. While there were 12 miRNAs (i.e., peu-miR169l, peu-miR394-3p, peu-miR6450, peu-miR399f, peu-miR6435, peu-miR169m, peu-miR6425-3p, peu-miR6440, peu-miR159d, peu-miR7834, peu-miR169j and peu-miR478b) detected in the stems, but not in roots, the last six miRNAs were also detected in the leaves. Five miRNAs (i.e., peu-miR399e, peu-miR399d, peu-miR6431, peu-miR6469, and peu-miR482c-5p) were only detected in the roots and not in the stems, among which three miRNAs (i.e., peu-miR6431, peu-miR6469, and peu-miR482c-5p) were also detected in the leaves. In addition, eight miRNAs (i.e., peu-miR481a, peu-miR7819, peu-miR172d, peu-miR474, peu-miR7826, peu-miR7823, peu-miR6456, and peu-miR7816) were detected in the leaves only.

### 2.3. Novel miRNAs in P. euphratica Roots and Stems

After identifying known miRNAs, the novel miRNAs were identified according to the criteria of miREvo and miRdeep2. In total, 101 novel miRNAs were identified, and 59 out of the 101 novel miRNAs contained the complementary miRNA* sequences (Table S5). The length, negative minimal folding free energies (MFE) values, minimal folding free energies index (MFEI) values, hairpin structure and the first nucleotide bias of novel miRNAs were counted. The lengths of mature novel miRNAs and their pre-miRNA sequences were 18 to 24 nt and 38 to 292 nt, respectively. The negative MFE values varied from −127.0 to −8.8 kcal/mol, with an average value of –49.4366, which was less than those of the rRNA (–33 kcal/mol) and tRNA (–27.5 kcal/mol). MFEI values ranged from 0.63 to 2.93; the average value was 1.108, higher than that of the tRNA (0.64) and rRNA (0.59) [47]. A total of 48.5% of the first nucleotide bias tendency was U in novel miRNAs. This result agrees with the proposal that AGO1 harbors miRNAs that preference a 5′ terminal U. In addition, all data indicates that these novel pre-miRNAs have a high hairpin structure stability (Table S5).

Furthermore, 87 of the 101 novel miRNAs were shared in roots, stems, and leaves (Figure 2B, Table S5). Similar to known miRNAs, some novel miRNAs also showed tissue specificity, with seven novel miRNAs specific to stems and roots (Table 3) and some others found in leaves [39]. Five novel miRNAs (i.e., miR-n22, miR-n49, miR-n51, miR-n55, miR-n70) were detected in both stems and leaves, but not in roots. Two novel miRNAs (i.e., miR-n17, miR-n56) were identified in the roots and leaves, and miR-n99 was identified in roots only.

### 2.4. Differential miRNA Analysis between the Leaf, Root, and Stem of P. euphratica

**Figure 3:**
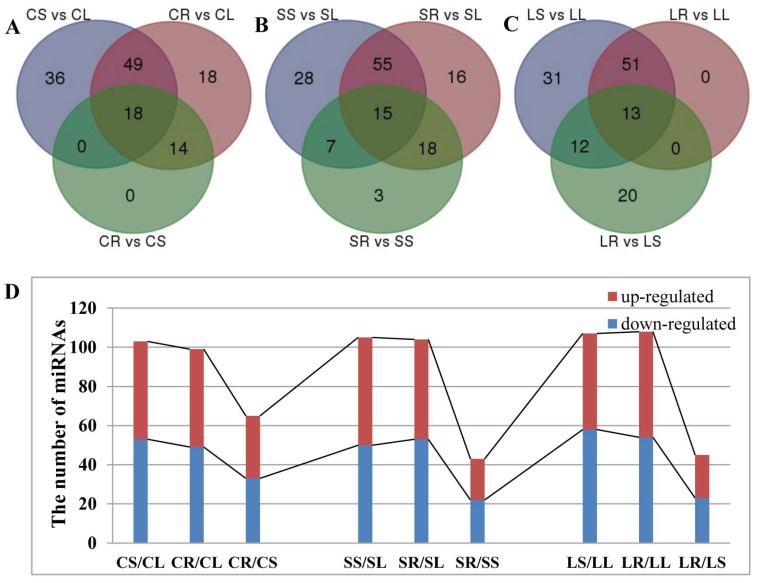
Significant differentially expressed miRNAs across the leaves, roots and stems in *P. euphratica*. (**A**) Venn diagram of significant differentially expressed miRNAs in the control groups of leaves, roots and stems. (**B**) Venn diagram of significant differentially expressed miRNAs in the one-day-treatment groups of leaves, roots, and stems. (**C**) Venn diagram of significant differentially expressed miRNAs in the four-day treatment groups of leaves, roots and stems. (**D**) The number of up- or downregulated miRNAs between each comparison in the different tissues.

The analysis of known and novel miRNAs showed that miRNAs exhibited a differential pattern in different tissues. Thus, different miRNAs from the leaf, root, and stem of *P. euphratica* were further analyzed. A total of 191 miRNAs, including 109 known miRNAs and 82 novel miRNAs, showed differential expression between the leaf, root, and stem of *P. euphratica* (Figure 3, Table S6).

In the control libraries of CL, CS, and CR, there were 83 known miRNAs and 52 novel miRNAs differentially expressed between the different tissues of *P. euphratica* (CS vs. CL, CR vs. CL, CR vs. CS). When compared with leaves, there were 103 miRNAs altered significantly in the stems, among which 53 miRNAs were downregulated, while 50 miRNAs were upregulated (CS vs. CL). In comparison with leaf tissue, there were 99 miRNAs significantly changed in the roots, with 49 miRNAs downregulated and 50 miRNAs upregulated (CR vs. CL). In addition, there were 65 miRNAs significantly altered, including 33 miRNAs downregulated and 32 miRNAs upregulated in the roots in comparison with stems (CR vs. CS) (Figure 3A,D and Figure S1, Table S6).

In one-day treatment libraries of SL, SS, and SR, there were 77 known miRNAs and 65 novel miRNAs differentially expressed between the different tissues of *P. euphratica* (SS vs. SL, SR vs. SL, SR vs. SS). When compared with leaves, there were 105 miRNAs altered significantly in the stems, among which 55 miRNAs were downregulated, while 50 miRNAs were upregulated (SS vs. SL). In comparison with leaf tissue, there were 104 miRNAs significantly changed in the roots, with 51 miRNAs downregulated and 53 miRNAs upregulated (SR vs. SL). In addition, there were 43 miRNAs significantly changed, including 21 miRNAs downregulated and 22 miRNAs upregulated in the roots in comparison with stems (SR vs. SS) (Figure 3B,D and Figure S1, Table S6).

In the four-day treatment libraries of LL, LS, and LR, there were 76 known miRNAs and 51 novel miRNAs significantly differentially expressed between the different tissues of *P. euphratica* (LS vs. LL, LR vs. LL, LR vs. LS). When compared with leaves, there were 107 miRNAs altered significantly in the stems, among which 49 miRNAs were downregulated, while 48 miRNAs were upregulated (LS vs. LL). In comparison with leaf tissue, there were 108 miRNAs significantly changed in the roots, with 54 miRNAs downregulated and 54 miRNAs upregulated (LR vs. LL). In addition, there were 45 miRNAs significantly changed, including 22 miRNAs downregulated and 23 miRNAs upregulated in the roots relative to the stems (LR vs. LS) (Figure 3C,D and Figure S1, Table S6).

### 2.5. Differentially Expressed miRNAs in Response to ABA in the Roots and Stems of P. euphratica

**Figure 4:**
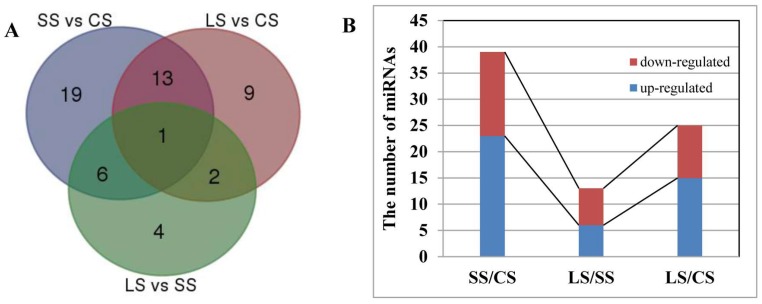
Significant differentially expressed miRNAs by ABA treatment in the stems of *P. euphratica*. (**A**) Venn diagram of significant differentially expressed miRNAs by ABA in the stems. (**B**) The number of up- or downregulated miRNAs responsive to ABA in the stems.

**Figure 5:**
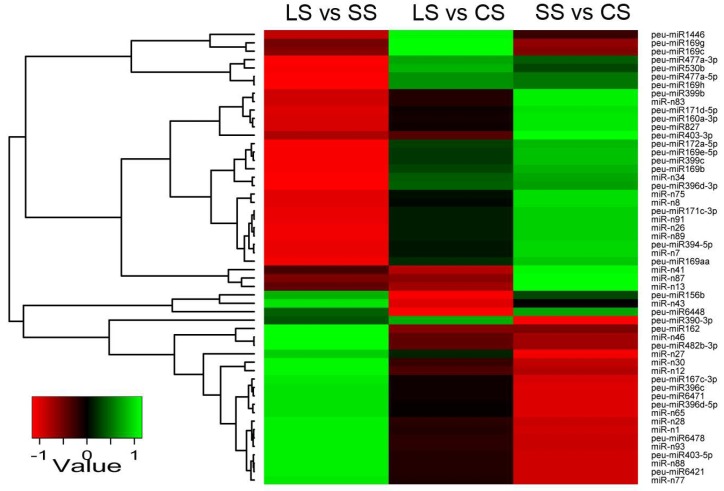
Hierarchical clustering analysis of differentially expressed miRNA in response to ABA in the stems of *P. euphratica*. The expression profiles were analyzed by Genesis software (http://genome.tugraz.at/) with hierarchical clustering method based on the transcripts per million (TPM) Log_2_FoldChange of miRNAs.

Fifty-four miRNAs containing 32 known miRNAs and 22 novel miRNAs showed up- or downregulated expression in the stems in response to ABA treatment (Figure 4A and Figure 5). Specifically, 39 miRNAs, including 23 upregulated and 16 downregulated, showed an altered expression pattern in the stems after one day of ABA treatment (SS vs. CS). Twenty-five miRNAs were upregulated (15 miRNAs) or downregulated (10 miRNAs) by a four-day ABA treatment (LS vs. CS). In addition, when compared with one-day ABA treatment, there were 13 miRNAs that responded to ABA with six upregulated and seven downregulated after a four-day ABA treatment (LS vs. SS) (Figure 4B). In general, with an increase in the time of ABA treatment, the proportion of upregulated miRNAs was higher than that of downregulated miRNAs in the stems.

**Figure 6:**
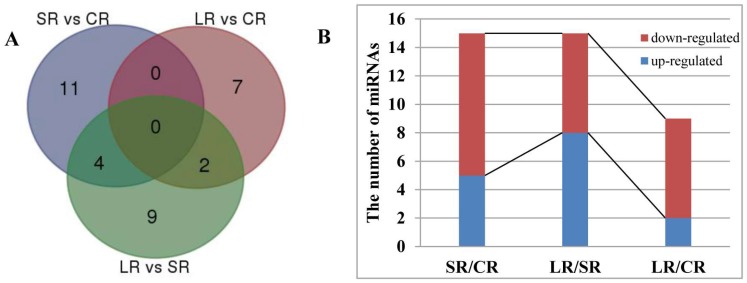
Significant differentially expressed miRNAs by ABA treatment in the roots of *P. euphratica*. (**A**) Venn diagram of significantly differentially expressed miRNAs by ABA in the roots. (**B**) The number of up- or downregulated miRNAs following ABA treatment in the roots.

**Figure 7:**
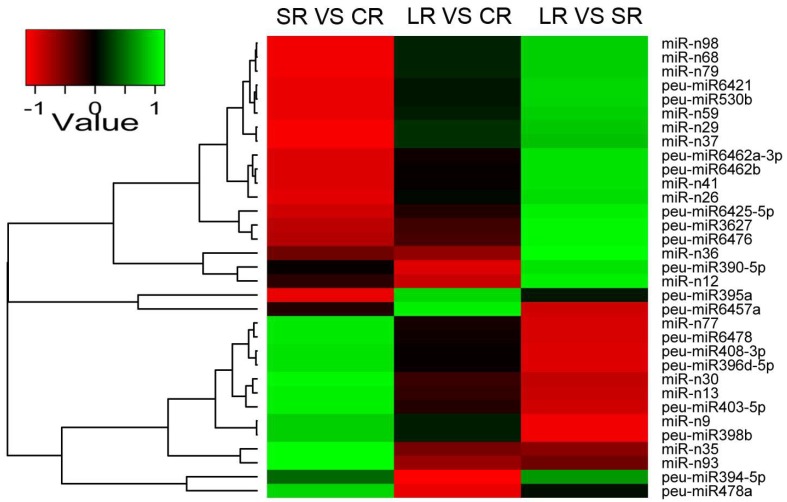
Hierarchical clustering analysis of differentially expressed miRNAs in response to ABA in the roots of *P. euphratica*. The expression profiles were analyzed by Genesis software (http://genome.tugraz.at/) with the hierarchical clustering method based on the TPM Log_2_FoldChange of miRNAs.

Similarly, the differential expression of miRNAs under ABA treatment in the roots was analyzed. A total of 33 miRNAs, including 17 known miRNAs and 16 novel miRNAs, showed altered expression in the roots in response to ABA treatment (Figure 6A and Figure 7). Specifically, 15 miRNAs in the roots were upregulated (five miRNAs) or downregulated (ten miRNAs) after one day of ABA treatment (SR vs. CR). Nine miRNAs were upregulated (two miRNAs) or downregulated (seven miRNAs) after four days of ABA treatment (LR vs. CR). When compared with one-day ABA treatment, 15 miRNAs were upregulated (eight miRNAs) or downregulated (seven miRNAs) by the four-day ABA treatment (LR vs. SR) (Figure 6B). Generally, with an increase in the time of ABA treatment, the proportion of downregulated miRNAs was higher than that of upregulated miRNAs in the roots, in contrast to the results obtained from stems, described above.

### 2.6. Validation of miRNAs by RT-qPCR

**Figure 8:**
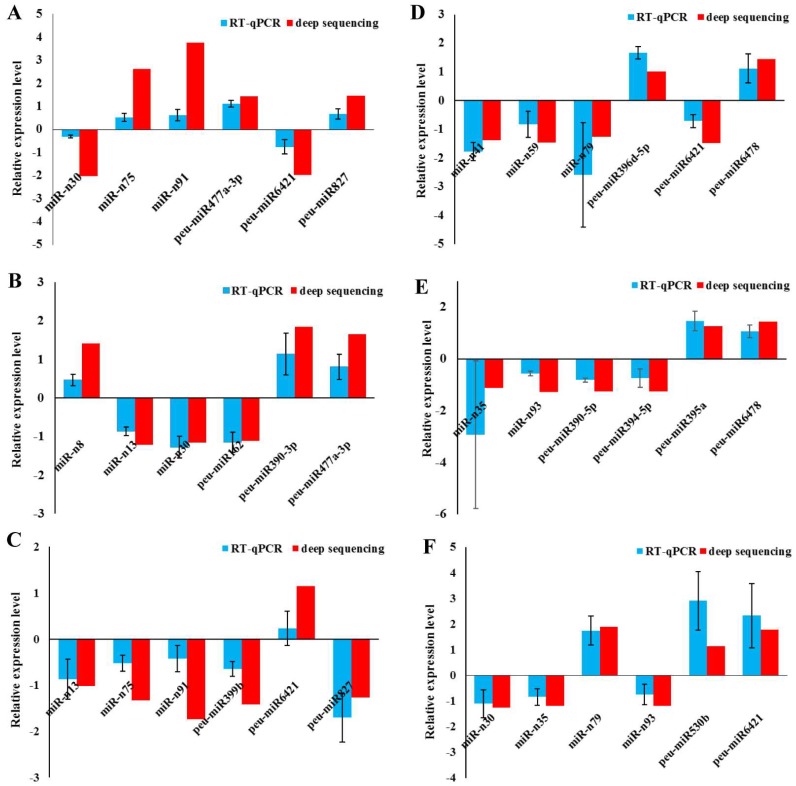
Verification of responses of miRNAs to ABA by real-time PCR in the stems (**A**–**C**) and roots (**D**–**F**). Differentially expressed miRNAs identified by high-throughput sequencing were confirmed by real-time qPCR, and their expression levels were compared between the three groups. The expression level of miRNA in deep sequences was performed with the R statistical software package; specifically, the “DESeq2” library was used with raw dates. The following comparisons of miRNA expression were completed: (**A**) SS vs. CS. (**B**) LS vs. CS. (**C**) LS vs. SS. (**D**) SR vs. CR. (**E**) LR vs. CR. (**F**) LR vs. SR.

To confirm the results of the significant differentially expressed miRNAs through high-throughput sequencing in the roots and stems, real-time qPCR analysis with three technical and three biological replicates was utilized, and six significant differentially expressed miRNAs— three novel miRNAs and three conserved miRNAs—were randomly selected for each comparison of SS/CS, LS/CS, LS/SS, SR/CR, LR/CR, and LR/SR. The results of all these miRNAs confirmed by RT-qPCR were consistent with the high-throughput sequencing analyses (Figure 8). This demonstrated that the results of the high-throughput sequencing were highly reliable.

### 2.7. The Prediction and Validation of miRNA Target Genes in P. euphratica

To better understand the regulatory mechanisms involved in the response of miRNAs to ABA, prediction and identification of their target genes was an important step. Firstly, a total of 3970 target genes were predicted for miRNAs with Targetfinder (Table S7). Then, degradome sequencing was conducted, and more than 15 million sequences were obtained. More than 99% of these raw reads could be mapped to the genome of *P. euphratica*. After processing and filtering the adaptor, contaminant, and repeat sequences, more than 6 million sequences of unique reads remained. Over 60% of these unique sequences were then matched to the genome of *P. euphratica* perfectly (Table 4). A total of 447 target genes were obtained by degradome sequencing (Table S8). Furthermore, CleaveLand was used to verify cleaved small RNA targets from degradome sequencing [48], and 447 miRNA-targeted transcript pairs, which were targeted by 125 miRNAs, were confirmed by degradome sequencing, among which 32 novel miRNAs were targeted at 133 genes, and 83 known miRNAs were targeted at 314 genes. Then the target transcripts were pooled and categorized into five categories based on the relative abundance of target gene reads. This showed that 139, 23, 132, 9, and 144 target genes were classified as being part of categories 1 to 5, respectively (Tables S8 and S9).

Furthermore, the target genes of ABA responsive miRNAs in the root were deeply analyzed. peu-miRNA477a-5p, targeted at *RGL1*, is involved in the GA signaling pathway [49]. peu-miR390, targeted at cytosolic NADP+-dependent isocitrate dehydrogenase, it appears to contribute to NADPH production under oxidative stress, thereby participating in redox signalling linked to defense responses [50,51]. peu-miR-n68, targeted at *BAK1* (BRASSINOSTEROID INSENSITIVE 1-ASSOCIATED RECEPTOR KINASE 1-RELATED), is involved in the brassinosteroid (BR) signaling pathway [52]. peu-miRNA395a, targeted at *APS1* (adenosine 5′-phosphosulfate) [27], and peu-miRNA398, targeted at *CSD* (copper/zinc superoxide dismutase) [28], are involved in nutrition metabolism. peu-miRNA394-5p, targeted at *FBX6* (F-box protein), peu-miRNA530b, targeted at *SAP1* (stress-associated protein 1), and peu-miRNA408-3p [53] and peu-miRNA398 [54] are all involved in abiotic stresses. These results indicated that ABA responsive miRNAs also interact with other physiology pathways.

**Figure 9:**
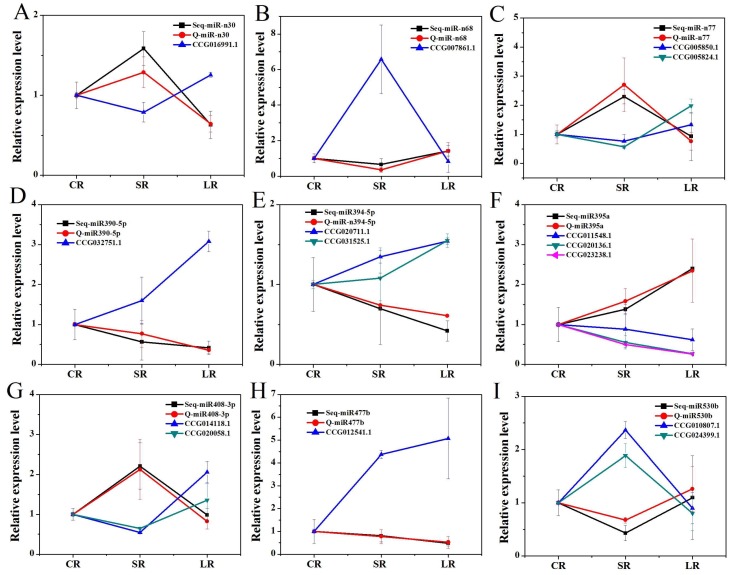
The expression profiles of predicted target genes and their corresponding miRNAs using RT-qPCR. The level of every gene in the control was set at 1.0. Error bars represent the standard deviation of three replicates. “Seq” means the results of high-throughput sequence, “Q” means the results of RT-qPCR. (**A**) The relative expression of miR-n30 with the predicted target CCG016991.4 (Potri.004G211300); (**B**) the relative expression of miR-n68 with the predicted target CCG007861.1 (Potri.009G090700); (**C**) the relative expression of miR-n77 with the predicted targets CCG005850.1 (Potri.005G225600) and CCG005824.1 (Potri.005G171700); (**D**) the relative expression of miR390-5p with the predicted target CCG032751.1 (Potri.010G176000); (**E**) the relative expression of miR394-5p with the predicted targets CCG020711.1 (Potri.001G057100) and CCG031525.1 (Potri.003G171300); (**F**) The relative expression of miR395a with the predicted targets CCG011548.1 (Potri.008G159000), CCG020136.1 (Potri.012G001400) and CCG023238.1 (Potri.007G108900); (**G**) the relative expression of miR408-3p with the predicted targets CCG014118.1 (Potri.002G188000) and CCG020058.1 (Potri.014G049600); (**H**) the relative expression of miR477b with the predicted targets CCG012541.1 (Potri.012G093900) and CCG030622.1 (Potri.015G091200); and (**I**) the relative expression of miR530b with the predicted targets miR530b CCG010807.1 (Potri.014G099700) and CCG024399.1 (Potri.010G076700).

In order to validate the effectiveness of our bioinformatics pipeline, the expression levels of predicted targets were measured by RT-qPCR to study whether the predicted target genes were actually regulated by corresponding miRNAs. Nine miRNAs and their predicted target genes were randomly selected. It can be observed that peu-miR-n30, peu-miR-n77, and peu-miR408-3p were upregulated with ABA for one day and downregulated for four days, and their target genes were all downregulated with ABA for one day and upregulated for four days. peu-miR390-5p, peu-miR394-5p, and peu-miR477b were downregulated with ABA treatment, and their target genes were all upregulated. peu-miR-n68 and peu-miR530b were downregulated with ABA treatment for one day and upregulated for four days, and their target genes were upregulated with ABA treatment for one day and downregulated for four days. Thus, the expression patterns of miRNAs using RT-qPCR are similar to the genome-wide analysis, and the expression profiles of miRNAs and their target genes were complementary (Figure 9).

## 3. Discussion

**Figure 10:**
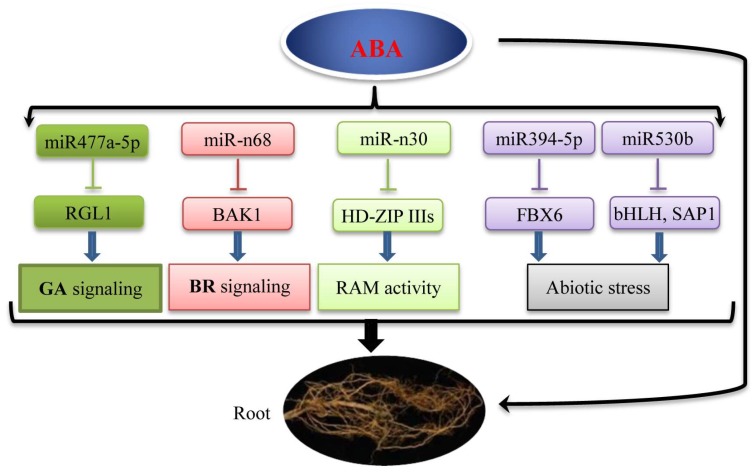
A new model of ABA regulation of root growth through miRNA-mediated pathways. The arrow indicates positive regulation. The inverted “T” denotes the interactions among miRNA-target genes predicted and investigated in this study; the “arrows” mean “involved in”. RGL1: repressor of GA1-like (Potri.012G093900); BAK1: BRASSINOSTEROID INSENSITIVE 1-ASSOCIATED RECEPTOR KINASE 1-RELATED (Potri.009G090700.1); HD-ZIP III: Class III Homeodomain Leucine Zipper (Potri.004G211300); FBX: F-box protein (Potri.001G057100); bHLH: basic helix-loop-helix protein (Potri.014G099700); SAP1: A20/AN1 zinc-finger containing stress-associated protein 1 (Potri.010G076700).

Small RNAs, including miRNAs, are key regulators in different biological processes, such as plant growth, development, metabolic pathways, biotic stress, and abiotic stress [55]. It is well documented that miRNAs orchestrate different abiotic stress responses, including drought [36], cold [38], salt [37], and ABA [39] in poplar trees by high-throughput sequencing. ABA, as a stress response phytohormone, plays a vital role in the regulation of abiotic stresses, and much of the abiotic stresses can be mimicked by external application of ABA [56,57]. In addition, ABA can not only be synthesized in the root [10], but also acts as a necessary role in the maintenance of root growth during water stress [12]. In this study, based on previous studies on the ABA in the leaves of *Populus euphratica*, the physiological data analysis showed that the photosynthetic rate, stomatal conductance, and transpiration rate were significantly changed after one day of ABA treatment, and moderately recovered after four days of ABA treatment [39]. The roots and stems of *P. euphratica* were collected for this study. Many forms of differential expression of miRNAs in the root were identified following ABA (Figure 7). Furthermore, based on the ABA responsive miRNAs in the roots of *P. euphratica*, ABA regulated root growth and development through miRNA-mediated pathways were discussed regarding the following aspects (Figure 10).

### 3.1. peu-miRNA477 Involved in the Crosstalk between ABA and GA in Root Growth

peu-miRNA477, which targets *RGL1* (*repressor of GA1*-*like*, Potri.012G093900), which was confirmed by the degradome sequence, was one of the gibberellin negative regulatory factors (Table S8) [49]. The *rgl1* mutant plant became dwarfed in shoots, whereas it increased lateral root growth significantly, and also altered gibberellin and metabolite profiles in *Populus* [58]. With the expression level of peu-miRNA477a-5p increased in the stems (Figure 5, Table S3) and roots (Figure 7, Table S4) after the treatment of ABA, it acted as a negative regulatory factor and reduced the expression level of *RGL* (Figure 9), and then repressed shoot growth, whereas it promoted root growth in *P. euphratica*. Thus, it can be speculated that ABA repressed shoot growth and promoted root growth through a new pathway of repression of GA-induced elongation by miRNA477a-5p.

### 3.2. peu-miR-n68 Involved in the Crosstalk between ABA and BR in Root Growth

peu-miR-n68, a novel miRNA found in *P. euphratica*, which targets Potri.009G090700.1 (BAK1, BRASSINOSTEROID INSENSITIVE 1-ASSOCIATED RECEPTOR KINASE 1-RELATED) as predicted by Targetfinder (Table S7), was decreased after 1 day of ABA treatment, and then recovered to its normal levels after 4 days of ABA treatment (Figure 7). As we know, brassinosteroid (BR) signaling is essential for plant growth and development. A model of BRASSINOSTEROID INSENSITIVE1-mediated signaling in root growth has been established by a computational approach which predicts root growth solely on the basis of BRI1 receptor activity. The model faithfully predicts root growth, as has been observed in bri1 loss-of-function mutants [59]. BRI1-mediated signaling regulates normal cell cycle progression of root meristematic cells [60]. In *Solanum pimpinellifolium*, BRASSINOSTEROID INSENSITIVE1 is required for systemin-induced root elongation [61]. Biochemical interaction between BAK1 and BRL1/BRL3 (brassinosteroid insensitive1-like) is required for BR-mediated root growth [52]. Thus, with the decreased expression of peu-miR-n68 following ABA treatment in the roots of *P. euphratica* (Figure 7, Table S4), the expression of BAK1 was induced (Figure 9) and then promoted root growth.

### 3.3. peu-miR-n30-Mediated Target Genes Involved in RAM Activity in Root Growth

peu-miR-n30, a novel miRNA found in *P. euphratica* which targets HD-ZIP IIIs (Potri.004G211300) as predicted by Targetfinder (Table S7), was increased after 1 day of ABA treatment, and then recovered to its normal levels after 4 days of ABA treatment (Figure 7). A previous study showed that overexpression of miR166/165 promotes the activity of root apical meristems (RAM), while its target HD-ZIP IIIs confers reduced RAM activity [62]. HD-ZIP III factors also appeared to determine both root growth rate and meristem size. Overexpression of HD-ZIP IIIs leads to short roots and small root apical meristems, while mutants have long roots and large apical meristems [63]. In our study, the expression of peu-miR166 was also increased after ABA treatment, but not significantly. Thus, with the increased expression of peu-miR-n30 and peu-miR166 following ABA treatment in the roots of *P. euphratica* (Figure 7, Table S4), the expression of HD-ZIP IIIs was inhibited (Figure 9) and then promoted root growth by enhancing meristematic activity.

### 3.4. peu-miR394-5p and peu-miR530b Mediated Stress-Related Genes Involved in Root Growth

Plant root growth and development is greatly affected by different abiotic stresses. When the external water supply is deficient, the root architecture will be significantly altered to improve its water absorption efficiency [64,65]. The targeting of F-box protein genes (Potri.001G057100) by peu-miR394-5p was confirmed by the degradome sequence (Table S8), and the same results were also found in *Arabidopsis* [66]. F-box proteins play important roles in abiotic stresses and have been reported to be differentially regulated by abiotic stresses [67]. In rice, overexpression of the F-box protein gene also reduced the sensitivity of ABA and abiotic stress tolerance while promoting root growth [68]. Thus, with the declined expression of peu-miR394-5p in the roots response to ABA in *P. euphratica* (Figure 7), the target F-box protein genes were induced, which might confer abiotic stress tolerance. peu-miR530b, which increased first, and then declined, in the roots under ABA treatment (Figure 7), targeted the *bHLH* transcription factor (Potri.014G099700) (Table S8). Overexpression of *bHLH122* would improve the endogenous ABA content in cells and promote root growth [69]. In addition, A20/AN1 zinc-finger containing stress-associated proteins (*SAP1/11*, Potri.010G076700) was another predicted target gene of peu-miR530b (Table S8). Many studies have reported that *SAP1/11* confers improved resistance to abiotic stresses in various plants. Overexpression of *OsSAP1* in tobacco confers increased tolerance to abiotic stresses [70]. Enhanced tolerance to ABA and salt stress is regulated by *OsSAP8* in *Oryza sativa* [71]. Therefore, peu-miR530b might participate in the response of ABA, as well as the tolerence of abiotic stresses mediated by *bHLH* or *SAP1/11* in *P. euphratica*.

Above all, our functional analysis of these miRNAs and their targets revealed a new model for the ABA regulation of root growth through the miRNA-mediated pathway. In particular, it showed that ABA crosstalk with other phytohormones, such as gibberellic acid, brassinosteroid, and auxin, in the regulation of root growth and development through miRNA-mediated pathways. Our results support previous conclusions [6]. More evidence is needed to further support our results.

## 4. Materials and Methods

### 4.1. Plant Materials and ABA Treatment

One-year-old *P. euphratica* plantlets were acquired from the Xinjiang Uygur Autonomous Region of China, and three plants with similar heights were planted in individual 5 L pots containing loam soil and placed in a greenhouse at Beijing Forestry University. After natural growth from April to July, an aqueous solution with or without 300 μM ABA was used to water the *P. euphratica* plants. For the treated groups, 1 L 300 μM ABA solutions were irrigated, and pure water, instead of ABA solution, was applied as the control group. Each treatment group had three individual pots as biological replicates. Stems collected one day after ABA treatment were defined as the short-term ABA treatment group (SS). Those collected four days after ABA treatment were defined as the long-term ABA treatment group (LS), and the control group (CS). Root tissues were also collected at the same time, and were named as SR, LR, and CR, respectively. All the samples in this study were collected at the same time during the day. To reduce error, three independent biological replicates were used for each stage. All of the samples were immediately frozen in liquid nitrogen and stored at –80 °C until use.

### 4.2. High-Throughput Sequencing of Small RNA

Total RNA was extracted from 18 samples of CS, SS, LS, CR, SR, and LR using the CTAB method [72,73]. RNA quality and quantity was examined using an Agilent 2100 Bioanalyzer with the RNA 6000 Nano Kit (Agilent Technologies, Santa Clara, CA, USA). Sequencing libraries were generated using NEBNext^®^ Multiplex Small RNA Library Prep Set for Illumina^®^ (NEB, Ipswich, MA, USA) according to the manufacturer’s specifications. Then, Illumina HiSeq technology was used for high-throughput sequencing (Illumina, San Diego, CA, USA).

### 4.3. Sequencing Data Analysis for miRNA Identification and Annotation

Low-quality reads were removed from raw sequencing data, and 10–30 nt sRNAs were filtered without more than 10 nt single nucleotide repeats, or 5′ adapter contaminants, or more than 10% poly N, or with a 3′ adapter on the insert tag. Then, bowtie (http://sourceforge.net/projects/bowtiebio/files/) was used to map the clean sequencing reads to the genome of *P. euphratica* [40] without any mismatch. All mapped sRNAs which were previously discovered and registered in miRBase (Release 21 http://www.mirbase.org/) by the BlastN algorithm with both mature and hairpin without any mismatches in *P. trichocarpa* were annotated as known miRNAs. The remaining reads, which can be mapped to GenBank (http://www.ncbi.nlm.nih.gov/ genbank/) and Rfam (11.0 release, http://rfam.xfam.org/) database, were annotated as noncoding RNAs (i.e., rRNAs, tRNAs, snRNAs, scRNAs, and snoRNAs). Further, after removing the repeat-associated RNAs (Repbase v.18.07, http://www.girinst.org/) and nat-siRNAs (*P. trichocarpa* in PlantNATsDB, http://bis.zju.edu.cn/pnatdb/), miREvo and miRdeep2 were utilized to predict novel miRNAs; all novel miRNAs candidates had to meet the following criteria: (i) both the miRNA and miRNA* (miRNA complementary sequence) were covered by sRNA-sequencing reads; and (ii) in cases without miRNA*, candidate miRNAs had to be identified in multiple and independent libraries [74,75]. The secondary structure, the minimal folding free energies (MFE) and the minimal folding free energies index (MFEI) were also analyzed as described previously [74,76].

### 4.4. Differential Expression Analysis of miRNAs Response to ABA in the Roots and Stems

To analyze the differential expression of miRNA, miRNA expression was normalized to calculate the expression of transcripts per million (TPM) in each library. Differentially expressed known and novel miRNAs were compared using the “DEGseq2” library in R statistical software among different samples from the stems and roots [77]. The fold-changes and *p*-values were calculated from the normalized expression. Significantly differential expression was defined as |log_2_ratio| ≥ 1 and an adjusted *p*-value ≤ 0.05 [78]. Transcripts per million (TPM) was defined as normalized expression = (number of miRNA reads/total number of clean reads) × 1,000,000 [79]. The heat maps of expression profiles were analyzed with Genesis software (http://genome.tugraz.at/) with a hierarchical clustering method based on the TPM Log_2_FoldChange of miRNAs [80].

### 4.5. RT-qPCR Validation

To confirm the results of high-throughput sequencing, six miRNAs, including three novel miRNAs and three conserved miRNAs, were randomly selected for RT-qPCR for each comparison. The CTAB method was used to extract RNAs from each sample [72,73]. Mature miRNA reverse transcription was performed by using the miRNA First-Strand cDNA Synthesis Kit (Aidlab Biotechnologies, Beijing, China). A miRNA Real-Time PCR assay kit (Aidlab Biotechnologies, Beijing, China) was used for the RT-qPCR analysis. Briefly, 20 μL reaction volume with 0.5 μL cDNA, 0.4 μM of each primer, 10.0 μL of 2× miRNA qPCR Mix, and 8.7 μL ddH_2_O was mixed in 96-well plates with a StepOne Plus PCR System (Applied Biosystems, Foster City, CA, USA) under the following default cycling conditions (40 cycles of 95 °C for 10 s, 60 °C for 20 s, and 72 °C for 30 s). All of the reactions were performed in triplicate. peu-5.8s rRNA was used as an internal control for miRNA [81]. To further confirm the relationships between miRNAs and their target genes, *UBQ* was used as an internal control for target genes, and the 2^-ΔΔCT^ method was adopted to calculate the relative expression of RT-qPCR [82]. All the primers used in this study are listed in Table S1.

### 4.6. MiRNAs Targets Prediction by TargetFinder and Degradome Sequencing

TargetFinder (http://targetfinder.org/) was used to predict the target genes of miRNAs [83,84]. A penalty score criterion was used according to the alignment between each miRNA and its potential target as follows: (1) G:U mismatch penalty, 0.5 points; (2) other mismatch penalty, 0.5 points; and (3) if the above two cases occurred at the 2nd to 13th bases, double the penalty was given. A score of four points was used as the screening criteria.

In order to obtain as much degradome data as possible, a mixed sample containing equal amounts of total RNAs from each of the 18 samples was used for degradome sequencing using the PARE protocol [85] with the following steps: (1) the biotinylated random primers were mixed with the RNA sample; (2) RNA fragments containing biotin random primers were captured by magnetic beads; (3) 5′-adapters were attached to the RNA fragments; (4) cDNA libraries were obtained by reverse transcription; and (5) PCR amplification was performed and the libraries were established. Then, degradome sequencing was performed with an Illumina Hiseq 2500. Fastx-Toolkit to remove the adapters and low-quality nucleotide reads from the raw sequencing data. The remaining clean reads were further analyzed by Cleaveland Pipeline 2.0 software with default parameters [48,86]. The genome of *P. euphratica* [40] was used as a reference genome for mapping the sequencing reads. The National Center for Biotechnology Information (NCBI) database and PopGenIE (http://popgenie.org/) were also used to predict functions of the target genes of miRNAs.

Further, the following criteria were used to divide the cleaved target transcripts into five categories: (1) the abundance of raw reads at the cleavage site is the maximum on the transcript and there is only one maximum; (2) the abundance of raw reads at the cleavage site is the maximum on the transcript and there is more than one maximum; (3) the abundance of reads at the cleavage site is not the maximum, but is equal to or higher than the median for the transcript; (4) the abundance of reads at the cleavage site is less than the median for the transcript; and (5) the abundance of reads at the cleavage site has only one match with the transcript.

### 4.7. Accession Number

The sequenced data from the roots and stems of *Populus euphratica* obtained in this work have been submitted to the Gene Expression Omnibus (GEO) database of the NCBI, and the accession number is GSE107823. The leaves’ data were obtained from the Sequence Read Archive, and the accession number is SRP077948 [39].

## 5. Conclusions

We constructed eighteen high quality sRNA libraries based on *P. euphratica* stems and roots for high-throughput sequencing. In total, 255 unique mature sequences which contained 154 known miRNA and 101 novel miRNAs were identified. There were 191 miRNAs, including 109 known miRNAs and 82 novel miRNAs with differential expression between the leaves, roots and stem. There were 54 miRNA responses to ABA in the stems, including 32 known miRNA and 22 novel miRNAs, and a total of 33 miRNA responses to ABA in the roots, with 17 known miRNA and 16 novel miRNAs. In addition, 3970 target genes were predicted by Targetfinder, and 447 target genes from 15 million degradome sequences were obtained. Furthermore, our genome-wide analysis of these miRNAs and their targets revealed a new model of ABA regulation of root growth through a miRNA-mediated pathway. This pathway features peu-miR-n68, and peu-miR477a-5p which are involved in the crosstalk between ABA and other phytohormones. Above all, our results provide new insight into the mechanisms of ABA’s involvement in root growth through miRNA-mediated pathways.

## Figures and Tables

**Table 1:** Deep sequencing reads for sRNAs of *P. euphratica* stems and roots.

Sample	Raw Data	Clean Reads	Length Filtered Reads	Mapped Reads	Length Filtered Unique Reads	Mapped Unique Reads
CS1	15,000,000	14,587,199 (97.25%)	8,096,532	4,932,121	3,046,077	1,258,568
CS2	12,295,483	11,416,579 (92.85%)	5,998,393	2,391,734	3,216,544	855,550
CS3	16,028,756	15,256,023 (95.18%)	9,304,524	5,142,929	2,672,612	681,844
SS1	12,314,579	12,051,738 (97.87%)	4,489,652	2,836,870	1,742,854	722,719
SS2	12,235,671	11,787,281 (96.34%)	4,253,676	2,551,136	1,766,408	720,158
SS3	10,721,576	10,189,259 (95.04%)	3,307,671	1,850,502	1,428,834	539,275
LS1	10,964,853	10,620,198 (96.86%)	3,939,212	2,362,697	1,415,467	521,575
LS2	11,332,951	10,519,899 (92.83%)	1,440,580	659,872	580,173	156,926
LS3	16,245,725	15,452,361 (95.12%)	5,117,466	2,686,232	1,739,006	508,100
CR1	17,920,873	16,698,033 (93.18%)	9,605,828	1,555,617	3,616,174	334,181
CR2	18,537,368	18,101,660 (97.65%)	15,420,868	2,768,522	3,738,298	389,598
CR3	11,075,762	10,302,814 (93.02%)	5,578,796	1,395,324	2,345,761	347,958
SR1	11,588,883	10,762,002 (92.86%)	6,140,223	1,556,764	2,796,352	450,033
SR2	10,966,078	10,312,261 (94.04%)	8,254,705	2,095,221	3,544,129	538,110
SR3	11,945,678	11,430,601 (95.69%)	5,562,200	1,871,703	1,778,115	345,995
LR1	12,971,014	12,601,308 (97.15%)	6,650,163	3,399,255	2,706,102	777,214
LR2	14,093,468	13,780,786 (97.78%)	10,598,000	1,733,908	3,047,028	240,787
LR3	10,769,268	10,361,201 (96.21%)	5,914,549	1,497,009	1,906,663	234,996

CS: control groups of stems without abscisic acid (ABA) treatment. SS: stems collected one day after ABA treatment were defined as the short-term ABA treatment. LS: stems collected four days after ABA treatment were defined as the long-term ABA treatment (LS). CR: the control groups of roots without ABA treatment. SR: roots collected one day after ABA treatment were defined as the short-term ABA treatment. LR: roots collected four days after ABA treatment were defined as the long-term ABA treatment. The numbers, of 1–3 mean three replicates.

**Table 2:** Known miRNA specially detected in the stems and roots of *P. euphratica* (not in leaves).

miRNA	Sequence (5′-3′)	LM (nt)	Reference miRNA	Family
peu-miR169l	AAGCCAAGGAUGACUUGCCUG	21	ptc-miR169o	
peu-miR169m	UAGCCAAGGAUGACUUGCUCG	21	ptc-miR169x	MIR169_1
peu-miR171f	GGAUUGAGCCGCGCCAAUAUC	21	ptc-miR171k	MIR171_1
peu-miR394-3p	CUGUUGGUCUCUCUUUGUAA	20	ptc-miR394a-5p	MIR394
peu-miR399d	UGCCAAAGGAGAUUUGCCCCG	21	ptc-miR399a	MIR399
peu-miR399e	UGCCAAAGAAGAUUUGCCCCG	21	ptc-miR399d	MIR399
peu-miR399f	UGCCAAAGGAGAGUUGCCCUA	21	ptc-miR399i	MIR399
peu-miR477c	GGAAACCUUUUGUGGGGGUUUG	22	ptc-miR477c	MIR477
peu-miR6435	UGAAUAAUGGAGACACUCUAG	21	ptc-miR6435	
peu-miR6450	CGAACACAGGACUCAAGGCUA	21	ptc-miR6450b	
peu-miR6472	UAGUGAAUUCUAGGUCUCAAUC	22	ptc-miR6472	

LM: length of mature miRNA.

**Table 3:** Novel miRNA especially detected in stems and roots of *P. euphratica* (not detected in leaves).

miRNA	Sequences	miRNA*	Arm	LM(nt)	Location	Stand	MEF	LP(nt)	GC%	MEFI
miR-n95	uuauuuaaauuugauuucuuu	No	3p	21	scaffold35.1: 322060..322378	+	−26.3	62	9.68%	4.38
miR-n96	uuggaggaaauauauuuuggc	Yes	3p	21	scaffold4.1: 1609767..1610085	−	−38	84	38.10%	1.19
miR-n97	ugaagagguagagaguguaauu	Yes	5p	22	scaffold476.1: 74348..74667	+	−67.7	146	47.26%	0.98
miR-n98	gggacaaaaauggcauaagagg	No	3p	22	scaffold98.1: 88683..89002	−	−97.5	251	42.63%	0.91
miR-n99	aaggaaaaugcauagaacaagu	No	5p	22	scaffold32.1: 2021024..2021343	+	−20	46	30.43%	1.43
miR-n100	aauuuguacugugaaacu	No	5p	18	scaffold462.1: 47666..47981	+	−8.8	38	36.84%	0.63
miR-n101	uauagaugacuauauuuagggagc	Yse	5p	24	scaffold2579.1: 17957..18278	−	−84.9	192	32.81%	1.35

miRNA*: miRNA complementary sequence. LM: length of mature miRNA. LP: length of miRNA precursors. GC%: The percentage of the sum of guanine and cytosine. MEF: folding free energies. MFEI: the minimal folding free energies index.

**Table 4:** Data analysis of degradome sequencing in *P. euphratica*.

Sample	Total Reads	Ratio	Unique Reads	Ratio
Raw Reads	15,513,985	/	6,684,885	/
Mappable Reads	15,407,998	99.32%	6,634,914	99.25%
Transcript Mapped Reads	10,447,471	67.34%	4,224,109	63.19%

## Supplementary Materials

Supplementary materials can be found at http://www.mdpi.com/1422-0067/19/5/1481/s1.
