# Supplementary material for: Exploration of ABA Responsive miRNAs Reveals a New Hormone Signaling Crosstalk Pathway Regulating Root Growth of Populus euphratica
Source: Int J Mol Sci. 2018 May 16;19(5):1481. doi: 10.3390/ijms19051481 (PMC5983633; doi:10.3390/ijms19051481)
Supplement: Supplementary file 1 [file ijms-19-01481-s001.zip › Supplementary Figure S1.docx]

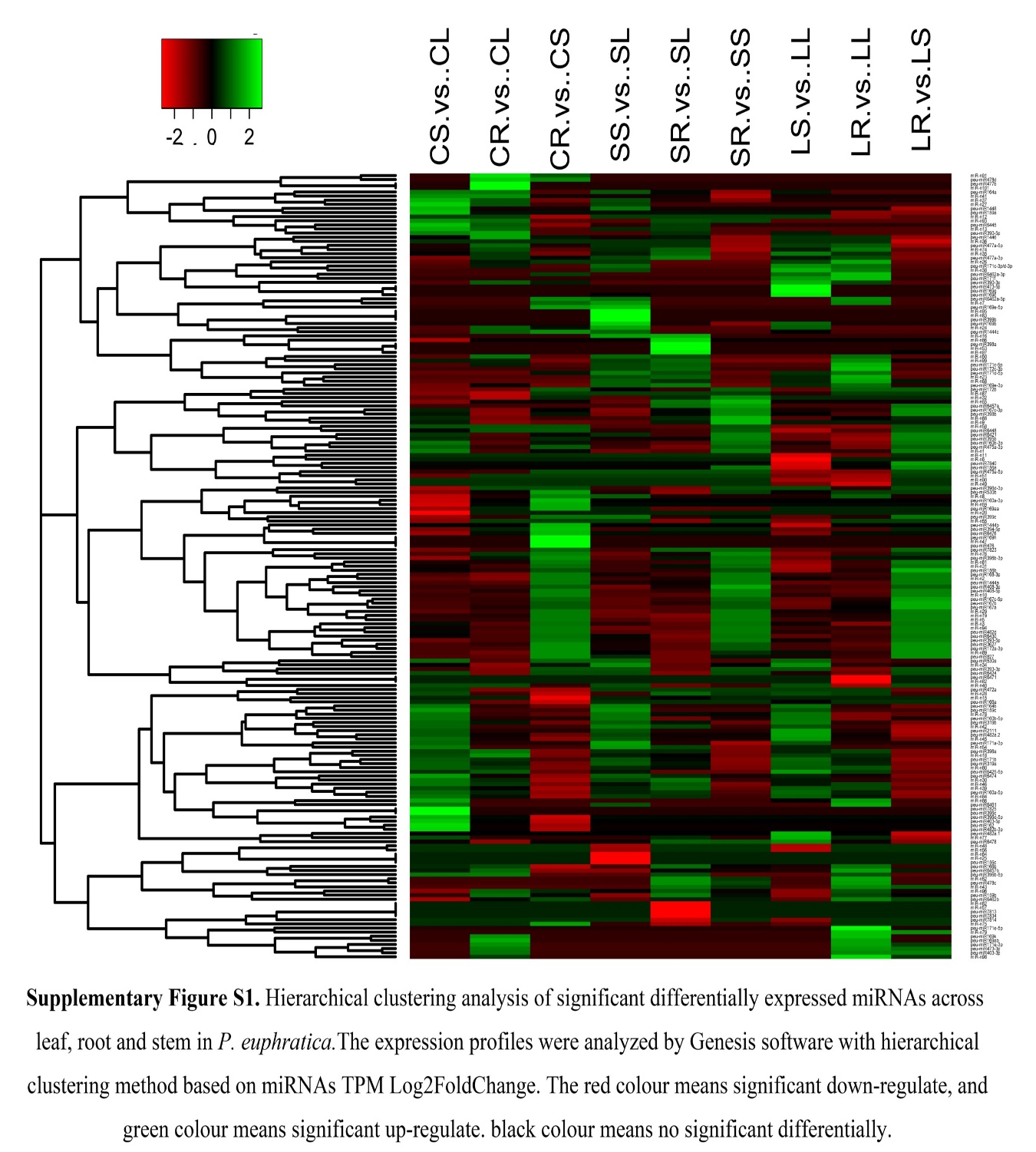


**Supplementary Figure S1.** Hierarchical clustering analysis of significant differentially expressed miRNAs across leaf, root and stem in P. euphratica.The expression profiles were analyzed by Genesis software with hierarchical clustering method based on miRNAs TPM Log2FoldChange. The red colour means significant down-regulate, and green colour means significant up-regulate. Black colour means no significant differentially.
